# Mechanobiology of MicroRNAs in Intervertebral Disk Degeneration

**DOI:** 10.26502/fjsrs0051

**Published:** 2023-01-17

**Authors:** Rajiv Supra, Devendra K. Agrawal

**Affiliations:** 1 College of Osteopathic Medicine, Touro University, Henderson, Nevada; 2 Department of Translational Research, College of Osteopathic Medicine of the Pacific, Pomona, California

**Keywords:** Apoptosis, Back pain, Degenerative disk disease, Extracellular matrix, Inflammation, low back pain, miRNA, Nucleus pulposus cells

## Abstract

Intervertebral disk degeneration (IDD) is an intricate pathological process contributing to one of the major causes of low back pain. The degradation of the extracellular matrix (ECM), inflammation, and apoptosis have all been investigated as critical factors involved in the pathology of degenerative disk disease. Additionally, the presence of aberrant microRNAs (miRNAs), conserved molecules that regulate the amount protein post-transcriptionally, may play a crucial role in the pathogenesis of IDD. Research regarding the dysfunction of miRNAs in IDD has been well researched over the past five years. Here, we provide a critical overview of the current knowledge of miRNAs, emphasizing the processes involved in the degenerative disk pathology.

## Introduction

One of the major causes of low back pain (LBP) is intervertebral disk degeneration (IDD) which has become a global burden greatly affecting the cost of healthcare. LBP affects 80% of adults at some point in their lives, becoming the leading cause of disability worldwide. The economic impact of LBP is vast costing up to 100 billion dollars per year in the United States [1,2]. The pathology of IDD is multifactorial consisting of age, lifestyle, epigenetics, and non-physiologic mechanical loading. The degeneration of connective tissue on the vertebrae occurs on a cellular level leading to gross physiological changes ultimately affecting spinal kinematics and decreased ability to bear compressive loads [2]. The underlying molecular mechanisms are still largely unknown; however, an increasing number of studies support that microRNAs (miRNAs) can influence many facets of cell activity including apoptosis, inflammation, and degradation of the extracellular matrix (ECM).

MicroRNAs, key mediators of gene expression, are a type of small noncoding RNAs that bind to the 3’-untranslated region inhibiting the translational process of specific mRNA molecules. Dicer is an enzyme that further processes mature miRNA in the cytoplasm after primary miRNA is generated in the nucleus. After incorporation in the RNA-induced silencing complex (RISC), they can suppress the translation of mRNAs ultimately regulating roughly 30% of human genes and cellular processes such as apoptosis and cytokine release [3]. MiRNAs have also been shown to interact with other RNAs such as circular RNAs, noncoding RNAs, and mRNAs, forming an intricate system of gene regulation in vertebral cells [4]. Dysregulation of these miRNA processes have been associated with intervertebral disk degeneration (IDD) [5]. This has motivated a new interest in miRNAs in relation to their role in IDD and potential new therapeutic approaches.

Several pathological mechanisms have been associated with IDD such as degradation of the ECM, cell apoptosis, and inflammation [6]. The ECM plays a critical role in the functionality of the intervertebral disk, consisting of type I and type II collagen providing much of the tensile strength [7]. The nucleus pulposus cells (NPCs), the cells that reside in the central region of the intervertebral disk, synthesize ECM components and dysregulation of this process results in the secretion of ECM degradative proteins such as matrix metalloproteinases (MMPs) [8]. Although this degradation process starts in the nucleus pulposus (NP), the annulus fibrosus (AF), the outer zone of the intervertebral disk, eventually becomes lost contributing to the overall degradation to the intervertebral disk [8,9]. Apoptosis and inflammation further enhance these degradative processes. It is well researched that NPCs release several cytokines such as interleukin (IL)-1β, IL-17, IL-6, and tumor necrosis factor (TNF)-α, which all can contribute to varying degrees of degeneration [10]. These cytokines have been deemed relevant because it is the source of pain associated with degenerative disk disease (DDD) through the infiltration into the nerve fibers and affecting their function [11].

**Figure 1:**
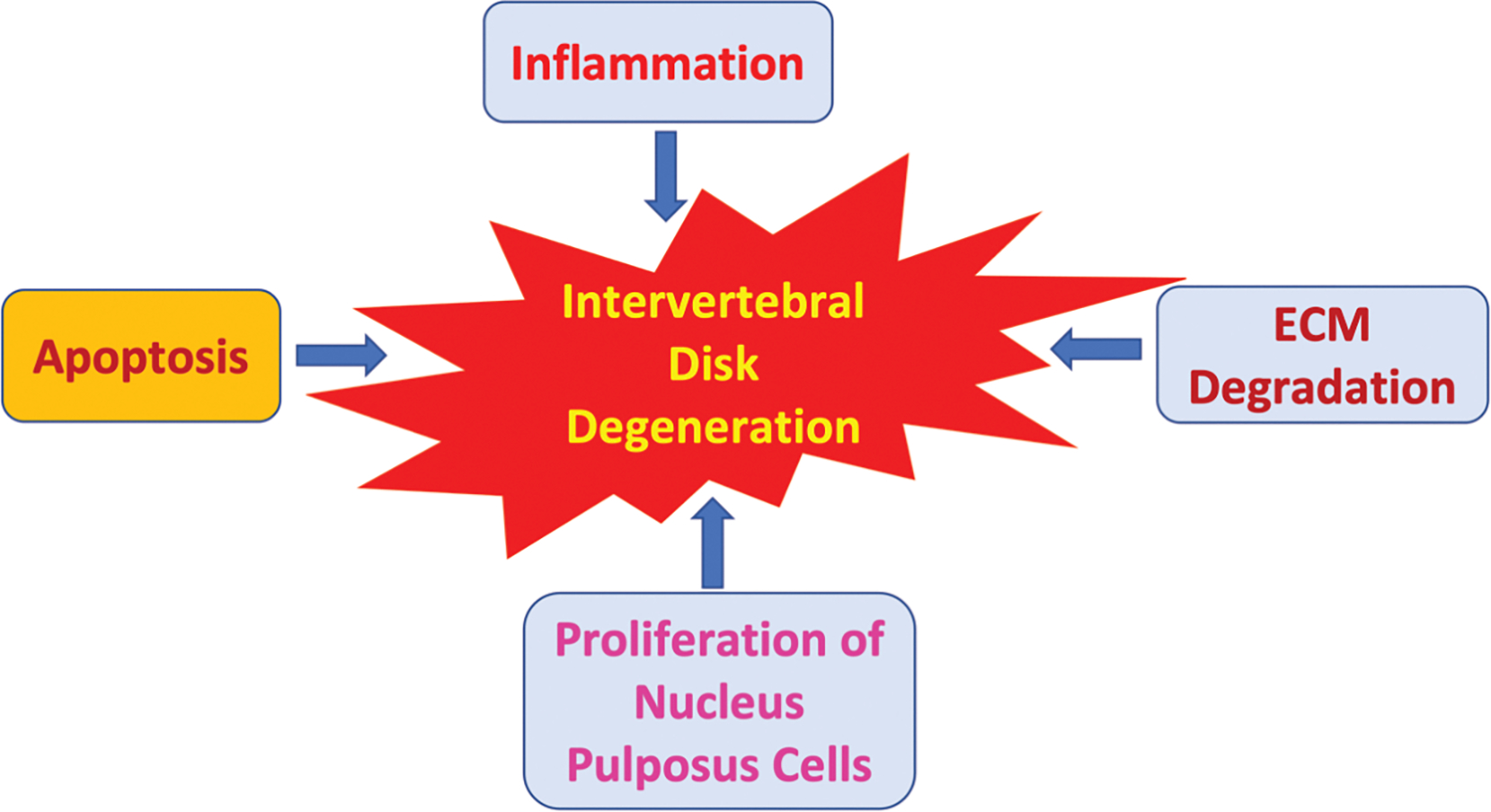
Effect of inflammatory processes, apoptosis, extracellular matrix (ECM) degradation, and proliferation of nucleus pulposus cells in the induction and acceleration of intervertebral disk degeneration.

The complex interplay between ECM degradation, inflammation, NP cell proliferation and apoptosis are the hallmarks of the DDD process [1] (Figure 1). Secretion of the inflammatory cytokines upregulates ECM degradative enzymes resulting in downregulation of ECM structure [12]. This degradation leads to a subsequent inflammatory response by NP cells as ECM fragments accumulate extracellularly [13]. Additionally, higher rates of apoptosis have been associated with decreased ECM production in IDD [14]. Based on the current research of the underlying mechanisms involved in IDD, standard therapy has been based on physiotherapy, pharmacological treatments, and arthroplasty [15]. Due to the high costs, limitations, and invasiveness of these treatments, there has been a high demand for novel therapies targeted at reducing pain and the degenerative process. Several therapies are being researched as potential alternatives which involves targeting miRNAs [16] (Table 1).

This review aims to provide a comprehensive overview of the role miRNAs play in the pathogenesis of degenerative disk disease. The pathology of IDD involving apoptosis, ECM degradation, inflammation, and nucleus populous cell function will be emphasized.

## MicroRNAs in Intervertebral Disk Degeneration

Ribonucleic acids are important in regulating genes. Among these, the miRNAs are a class of single stranded RNAs that function as post-transitional gene regulatory elements [17]. Their role in disk degeneration has been well documented involving the inflammation, NP cell apoptosis, and the ECM degradation processes [18]. NP cell apoptosis can have beneficial effects by offsetting aberrant NP cell proliferation during DDD while also being disadvantageous through the production of interstitial collagen fibers, weakening the tensile strength of the fibrous cap [19,20]. Thus, apoptosis of NP cells plays an integral role in DDD and tensile strength stability.

MiR-155 has been well-researched as one the miRNAs involved in regulating apoptotic pathways. Wang et al. revealed various miRNAs exhibiting differential expression in degenerative NP cells, specifically miR-155, which was the most downregulated [21]. When overexpressed, miR-155 inhibits NP cell apoptosis by suppressing Fas-associated protein with death domain (FADD) and caspase-3 expression. Additionally, when miR-155 is expressed in the cytoplasm of NP cells, there is an inverse relationship with FADD and caspase-3 [22,23]. MiR-27a has been another well-researched miRNA and its expression is high in NP cells. Its expression inhibits phosphoinositide-3 kinase (PI3K) by targeting its 3’-UTR and this inhibition was terminated by mutating miR-27a binding site [24]. In essence, when miR-27a is upregulated, apoptosis of NP cells occurs by targeting the PI3K pathway. Other miRNAs involved in the proliferation and degradation pathways of NP cells include miR-10b and miR-21. Previous studies have demonstrated the upregulation of miR-10b in degenerative NP tissues and overexpression of miR-10b increased NP cell proliferation through targeting homeobox D10 (HOXD10) [25]. MiR-21 has led to increased phosphorylation of Akt by targeting phosphatase and tensin homolog protein (PTEN), resulting in NP cell proliferation [26].

In addition to NP cell proliferation and degradation, miRNAs have a major impact on the ECM. The ECM is continuously degraded and synthesized by disk cells which are in a state of equilibrium [27]. This equilibrium becomes shifted towards degeneration in DDD through the alteration in the collagen type and proteoglycan content [28]. Chen et al. revealed the higher expression of miR-155 in lumbar spinal stenosis patients than those with lumbar disk herniation. Increased expression of miR-155 had a positive correlation with ligamentum flavum thickness and levels of type I and III collagen. miR-155 increased protein expression and mRNA of collagens type I and III in the fibroblasts of ligamentum flavum, while downregulation of miR-155 has the opposite effect [29]. Additionally, miR-377 has also been implicated in the pathologic remodeling of ECM. Protein kinase C (PKC) pathway has been shown to be a major regulator of chondrocyte differentiation. MiR-377 upregulation induced PKC signaling coupled with reductions in ADAMTS5, a metalloproteinase with thrombospondin motifs, and cleaved aggrecans [30]. Furthermore, it has been confirmed that miR-93 targets and regulates MMP-3, another collagen degrading enzyme. When downregulating miR-93, NP cells isolated from patients with vertebral disease led to increased levels of MMP-3 subsequently resulting in the degeneration of type II collagen [31]. Another study revealed MMP-13 overexpression in DDD [32]. MiR-27b takes part in this overexpression through downregulating NP cells isolated from degenerated disks [33]. Moreover, miR-133a downregulation observed in spinal tuberculosis and degenerative NP cells led to reduced levels of type II collagen expression [34,35].

Emphasizing miRNAs targeting against signaling pathways involved in ECM degradation, miR-98 was found to be involved in the IL-6/STAT pathway in DDD. When downregulating miR-98, IL-6 levels increased in NP tissue. Additionally, decreased levels of miR-98 initiated the STAT3 signaling cascade by increasing levels of MMP-2, pSTAT3, and STAT3, ultimately contributing to intervertebral disk degeneration [36]. MiRNA-132 and miR-7 have been shown to regulate the expression of growth differentiation factor 5 (GDF5). GDF5 is involved in ECM anabolism and polymorphisms of GDF5 resulted in degenerative diseases such as osteoarthritis [37–39]. Mi-R132 enhanced ECM degradation by targeting GDF5 leading to increased ADAMTS4 and MMP-13 expressions through the mitogen-activated protein kinase/extracellular signal-regulated kinases (MAPK/ERK) pathway. The miR-7 has also been studied enhancing ECM degradation by targeting GDF5, making miR-7 inhibition a promising therapeutic strategy [39].

Thus, findings from several investigations support the critical role of miRNAs in the metabolic pathogenesis of intervertebral disk degeneration. Variations in the miRNA expression of degenerated NP cells lead to dysregulation of metabolic enzymes leading to the compositional change of the ECM. This opens new avenues for potential therapeutic strategies which involves targeting the miRNAs involved in disk degeneration. For example, a preclinical study observed the in vivo changes of injecting an inhibitor of miR-141, a mRNA involved in disk degeneration, and studied its affects. This anti-miR-141 resulted in protective effects against IDD [40]. Further studies need to be conducted to evaluate the role of inflammation in the dysregulation of miRNAs and develop novel therapeutic strategies directed at the inflammation cascade.

## MiRNAs and Apoptosis

Normally, apoptosis maintains the stability of the internal environment of the cell through autonomous programmed cell death controlled by genes. There are exogenous and endogenous pathways that play a critical role in human NPC apoptosis. The density of NPCs in the intervertebral disk tissue is reduced through the FasL-Fas signaling pathway. This exogenous signaling pathway involves the Fas-related death domain-containing protein (FADD) and caspase-3 pathway [41]. A major endogenous apoptotic pathway consists of the anti-apoptotic protein B-cell lymphoma leukemia-2 (Bcl-2) family which originates in the mitochondria [42]. Furthermore, miRNAs have been shown to be implicated in cellular senescence changes in the expression profile of various miRNAs have been involved in the regulatory pathways leading to apoptosis in DDD [43,44].

MiR-185 and miR-143-5p are two well researched miRNAs involved in DDD. Studies were conducted using murine models with DDD [45]. The β-galactosidase binding protein involved in apoptosis, galectin 3, was targeted by miR-185 [46]. Rats with DDD had significantly higher levels of galectin 3 than the control group. When miR-185 was inhibited, these expression levels further increased. MiR-143-5p was upregulated in NP tissues of murine models. This was accomplished by eukaryotic elongation factor 2 (eEF2), one of the targets of miR-143-5p. Once targeted, the dysregulation of miR-143-5p led to decreased levels of eEF2 and activation of the adenosine monophosphate activated protein kinase (AMPK) pathway [47,48]. Activating the AMPK pathway subsequently reduced type II collagen and aggrecan levels and when miR-143-5p was inhibited, it resulted in lower levels of senescence through inactivating AMPK (48). Interestingly, NP cells recovered from patients suffering from disk disease exhibited higher levels of miR-143 and its upregulation was shown to increase apoptosis by targeting Bcl2 [49].

Another major miRNA involved in the apoptosis of NPCs is miR-138-5p. Wang et al. discovered that miR-138-5p was significantly upregulated in degenerated disks and its inhibition profoundly reduced apoptosis [50]. NPCs can be protected from excessive apoptosis by knocking out miR-138-5p. Protection from apoptosis was mediated through sirtuin 1 (SIRT 1) upregulation, which is induced by PTEN/PI3K/Akt pathways [51]. Activation of the AMPK signal pathway inhibited the differentiation of NPCs and promoted apoptosis. Additionally, miR-141 further progressed the process of DDD through targeting the SIRT1/NF-kB pathway, leading to apoptosis [52]. In vitro studies revealed knocking out miR-141 attenuated DDD by delivering the down-regulated miR-141 through nanoparticles in the DDD murine model. MiR-222 was also shown to be upregulated in degenerated NPCs and the overexpressed miR-222 activated Bax and caspase −3 but inhibited Bcl-2. Of note, activation of BCL-2 can inhibit apoptosis while activation of Bax and caspase-3 promotes apoptosis [53].

A recent study also revealed that miR-494 upregulation led to lower levels of SRY-Box Transcription Factor (SOX)-9, a gene that has been shown to protect against IL-1β-induced apoptosis [54]. MiR-494 downregulating SOX9 is seen in DDD, shedding light on the process of apoptosis in disk degeneration [55]. Additionally, targeting JunD and cytochrome c can lead to inhibition of miR-494 and may protect NPCs from TNF-α induced apoptosis [56]. Researchers have also identified increased levels of miR-494 in DDD murine models, whereas inhibitors of miR-494 led to increased Bcl-2 and neuro-oncological ventral antigen 1 (NOVA1) levels and reduced expression of caspase-3 and Bax [57]. MiR-494 is heavily involved in the apoptosis of NPCs and plays an integral role in DDD. The abnormal expression of miR-129 5p may also serve as a role in DDD. Studies have shown reduced levels of miR-129 5p in humans with DDD, whereas NPCs treated with miR-129-5-p and bone morphogenic protein 2 (BMP-2) silencing RNAs displayed improved survival and decreased apoptotic activity [58]. Similarly, miR-499a-5p also exhibited significant downregulation in human degenerated NP cells. Knocking out miR-499a–5p enhanced NPC apoptosis, MMP-13, and MMP-3 expression while decreasing aggrecan and type II collagen levels. Furthermore, overexpressing miR-499a–5p reduced apoptosis in TNF-α treated NPCs. The abnormal expression of SOX4, however, weakened the negative of miR-499a–5p on apoptosis of NP cells [59]. This suggests that miR-499a–5p may be influenced by targeting SOX4.

A deeper understanding of the apoptotic processes involved in DDD is needed and may provide new therapeutic approaches in delaying or possibly reversing DDD. Another component that is heavily involved in DDD is inflammation and further research can lead to the development of novel therapies in reducing DDD.

## Inflammation

Numerous studies have revealed inflammation as a key factor in the process of DDD. Inflammatory mediators such as ILs (interleukins), tumor necrosis factor (TNF)-α, nitric oxide, and prostaglandin E2 (PGE2) are the main regulators of the inflammatory response within the intervertebral disk [60]. Studies reveal that miRNAs can accelerate or delay the process of DDD through regulating inflammatory cytokines such as IL-1β and TNF-α [61].

MiR-146a was reported to inhibit mRNA expression of IL-1 β-mediated catabolic proteinases and MMPs [62]. However, Lv et al. revealed in peripheral mononuclear cells of patients with degenerative disk disease, miR-146 was significantly downregulated [63]. Additionally, they discovered that overexpression of miR-146a downregulated levels of TNF- α, IL-6, and IL-1β in lipopolysaccharide-stimulated NPCs. MiR-194-5p was also found to be downregulated in patients with intervertebral disk degeneration through miRNA microarray analysis [64]. Overexpression of miR-194-5p resulted in inhibiting and accelerating the expression of the cullin family genes 4A (CUL4A) and CUL4B. The inflammatory cytokines TNF-α and IL-6 also reduced levels of CUL4A and CUL4B in NPCs and AF cells. Similarly, miR-149 levels were significantly decreased in LPS-induced NPCs [64]. MiR-149 when overexpressed reduced the expression of collagen II and aggrecan and mitigated effects of MMP-3, ADAMTS4, and inflammatory mediators through targeting myeloid differentiation factor 88 [65].

MiR-27a was upregulated in the inflammatory DDD model using LPS stimulation. When miR-27a was inhibited, however, the p-p38/NF-kB expression and IL-6, IL-1, and TNF-a were decreased [66]. MiR-203-3p was upregulated in degenerated NP tissue and negatively correlated with estrogen receptor alpha (ERα) expression[67]. In the DDD murine model, miR-203-3p was shown to inhibit the inflammatory response and disk degeneration through targeting ERα. Activating TLR4/NF-kB pathways increased levels of inflammatory factors and expression of miR-625-5p while decreasing COL1A1 [68]. The rates of NPC apoptosis and TNF-α, IL-6, and IL-1 were also increased through the upregulation of miR-589-3p while levels of COL II and aggrecan were reduced through inhibiting Smad4 [69]. Interestingly, peripheral blood mononuclear cells from DDD patients revealed reduced expression of miR-146a while in the DDD murine model, miR-146a suppressed protein levels of TRAF6/NF-kB, leading to reduced levels of inflammatory cytokines in NPCs [70]. Collectively, the miRNAs that have been studied have a multitude affects contributing to inflammation, apoptosis, and ECM degradation seen in DDD (Table 1).

## Therapeutic Approaches in Degenerative Disk Disease (DDD)

**Figure 2:**
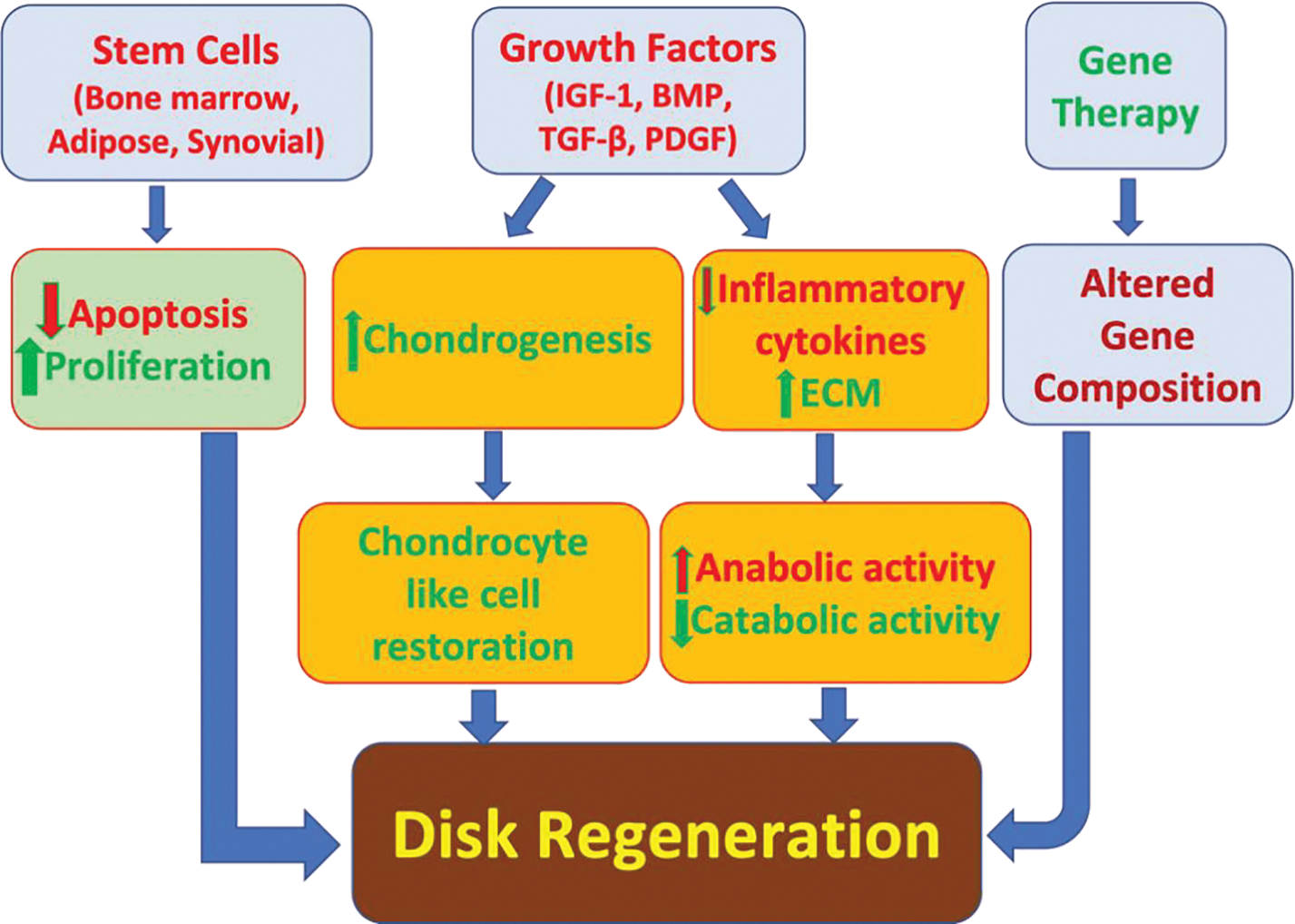
Current approaches in the treatment of degenerative disk disease. BMP, bone morphogenic protein; ECM, extracellular matrix; IGF-1, insulin like growth factor-1; PDGF, platelet derived growth factor.

Currently, there are several areas of growing research in novel strategies for DDD therapy (Figure 2). Genetic modification, stem cells, and growth factors all have shown beneficial effects in in vivo and in vitro studies [71].

Injecting growth factors into degenerated disks can stimulate the ECM and delay the degeneration process [72]. Growth factors such as bone morphogenic proteins (BMPs), insulin like growth factor-1 (IGF-1), platelet derived growth factor (PDGF), and transforming growth factor beta (TGF-β) are peptides that cause proliferation and differentiation of cells. They can stimulate anabolic function while reducing inflammatory cytokines such as IL-1, IL-6, and TNF-α. Growth factors are used for the restoration of DDD but are limited due to their biological half-lives [73,74]. The growth factors within platelet-rich plasma have been shown to be a promising therapeutic strategy for DDD [75]. In the rabbit DDD model, administering platelet-rich plasma with gelatin-based microspheres in the degenerated NPCs suppressed the degeneration process significantly. Administering platelet rich plasma in a similar rabbit model also revealed significant restoration of chondrocyte cells and disk height [76,77].

Gene therapy has been another growing field in the therapeutic strategies of DDD. This method involves introducing genes to target cells using viral and non-viral vectors. Target cells are then removed and put into culture medium where the cells can be altered and eventually re-implanted into target organs. The cells with genetic alterations can go on to produce proteins that support intervertebral disk regeneration [78]. Additionally, stem cells from adipose tissue, synovial tissue, and bone marrow can mediate changes in fibrocartilage like tissues. By transplanting cells, they can induce paracrine signaling to affect endemic cells to produce regenerative substances in intervertebral disks. De novo cells can also influence homeostasis and production of ECM proteins. These cells are obtained from embryonic human NPCs; mesenchymal stem cells (MSCs), and chondrocytes through autologous adipose tissue and bone marrow [79,80]. Adipose mesenchymal stem cells (ASCs) used on NPCs was shown to reduce expressions of TNF-α and IL-1β and decrease apoptosis. ASCs on AF cells increased proliferation and had anabolic effects while decreasing catabolic factors and inflammatory cytokines [81]. Collectively, gene therapy, stem cells, and growth factors have shown promising results in the regeneration of intervertebral disks, however further studies are needed to fully understand the limitations and applications of these approaches (Figure 2).

## Conclusion

MiRNAs are a critical component in regulating genes and are involved in the pathogenesis of degenerative disk disease. There is no optimal treatment for DDD, but substantial progress has been made in studying miRNAs and there association DDD. The studies currently available highlight miRNAs as a major factor in disk degeneration. Further studies are needed however, to research the impact miRNAs have on DDD and to develop new promising therapeutic strategies.

## Figures and Tables

**Table 1:** MiRNAs involved in degenerative disk disease

miRNA	Expression	Function	Target	Reference
miR-155	Decrease	Apoptosis	FADD	21
miR-27a	Increase	Apoptosis	PI3K	24
miR-21	Increase	NP Proliferation	PTEN	26
miR-377	Increase	ECM remodel	ADAMTS5	30
miR-93	Decrease	Collagen Degrade	MPP3	31
miR-27b	Increase	NP Proliferation	MMP-13	33
miR-133a	Decrease	Decrease Collagen	Type II Collagen	34, 35
miR-98	Decrease	ECM Degradation	IL-6/STAT	36
miR-132	Increase	ECM Degradation	GDF5	37, 38
miR-7	Increase	EMC Degradation	GDF5	39
miR-185	Increase	Apoptosis	Galectin 3	46
miR-143-5p	Increase	Reduce Type II Collagen	eEF2/AMPK	47, 48
miR-138-5p	Increase	Apoptosis	PTEN/PI3K/Akt	51
miR-141	Increase	Apoptosis	SIRT1/NF-kB	52
miR-222	Increase	Apoptosis	BAX/Caspase 3	53
miR-494	Increase	Apoptosis	SOX9	54
miR-129-5p	Increase	Apoptosis	-	58
miR-499a-5p	Decrease	Apoptosis	MMP-13	59
miR-146a	Increase	Inflammation	IL-1/MMP	62
miR-194-5p	Decrease	Inflammation	CUL4A/CUL4B	64
miR-149	Increase	Decrease Collagen II/Aggrecan	MyD88	65
miR-203-3p	Increase	Inflammation	ERa	67
miR-625-5p	Increase	Inflammation	TLR4/NF-kB	68
miR-589-3p	Increase	Decrease COL II/Aggrecan	SMAD4	69
miR-146a	Increase	Reduced Inflammation	TRAF6/NF-kB	70

## Data Availability

Not applicable since the information is gathered from published articles.
